# Rape in War Is Common, Devastating, and Too Often Ignored

**DOI:** 10.1371/journal.pmed.1000021

**Published:** 2009-01-27

**Authors:** The PLoS Medicine Editors

## Abstract

This month's Editorial discusses the unconscionable use of rape as a weapon of war and calls for more pressure to be put on international authorities to take concerted action and to make protection from sexual violence a central part of peacekeeping efforts.

Rape in war is by no means a new phenomenon, but its escalation as a deliberate, strategic, and political tactic is now undeniable. Most of its victims are women and girls, but men and boys suffer too. Whether isolated or systematic, rape's effects are devastating to individuals and damaging to whole communities. The physical consequences can include unwanted pregnancies, sexually transmitted infections including HIV, and genital injury including fistula, all of which can leave women scarred, disabled, unable to conceive, and deemed unsuitable for marriage [1,2]. The brutality of war rape is evident in genital mutilation, forced captivity, gang rapes in public or in front of family members, and rape with objects such as glass, sticks, gun barrels, and machetes [1,3]. Psychologically the effects are no less devastating. Traumatized by the event, women are often unable to care for their children or households, fear leaving their homes, can become socially ostracized and isolated, and may be rejected by their husbands, families, or communities [1–3].

First recognized as a problem internationally in the mid-1990s when “rape camps” that enslaved women and girls were discovered in the former Yugoslavia, systematic rape is now understood not as an unfortunate but inevitable by-product of war, but instead as a defining tactic of modern conflicts. Following the genocide in Rwanda, where an estimated 500,000 women were raped in 1994, a landmark decision by the International Criminal Tribunal recognized rape as a crime of genocide under international law [4]. Mass rape has been documented for recent conflicts in Bangladesh, Burma, Columbia, Liberia, Sierra Leone, and Somalia [5,6].

The toll of the ongoing conflict in the Democratic Republic of the Congo (DRC) is staggering: an estimated 5.5 million deaths [7], 1.5 million displaced people [8], and 500,000 victims of sexual violence [9] since 1991. Recent escalation of fighting has fuelled international press reports of a country more lawless than perhaps anywhere else on earth and where women are frequently and systematically victimized [10–13]. This “pandemic of sexual violence,” says Stephen Lewis, the former United Nations special envoy for HIV/AIDS in Africa, is “obscene,” “insanely savage,” and can only be described as “femicide” [10,14]. Dr. Denis Mukwege, the founder of the Panzi Hospital in eastern Congo that treats ten women survivors of rape every day, calls this war on women “the monstrosity of the century” [15]. Nonetheless, in areas of armed conflict rape is committed mostly with impunity and has been largely ignored by the international community.

As a terror tactic, rape aims to destroy or expel populations or ethnic groups, impregnate women, intimidate civilians, pillage land and resources, and may serve to increase military morale [16–18]. Husbands or family members, sometimes forced to watch, are also traumatized. In refugee camps in Darfur and Chad, where hundreds of thousands of Sudanese people are displaced, women are essentially imprisoned because they cannot even travel to get firewood or water without risking being raped [19]. In the DRC, different militia groups have distinct and recognizable ways by which they rape women, thus marking women with a signature that often mutilates and scars them for life, but also establishing the armed group's power and control. During the June 2008 UN meetings at which rape was classified as a weapon of war, the former UN peacekeeping commander Major General Patrick Cammaert summarized the effect of rape as a war tactic when he reported: “It has probably become more dangerous to be a woman than a soldier in an armed conflict” [20].

Health care professionals and humanitarian organizations working in conflict zones have long recognized the use of rape as a weapon of war, providing documentation from the ground that also reveals the absence of an adequate international response. Human Rights Watch reported this year that “women and girls continue to be brutally beaten and raped” by police, militia, and rebel groups in Darfur [21]. No progress has been made five years after the Sudanese government promised to combat sexual violence in the region and the UN–African Union peacekeeping forces were to mobilize security and protection for women. Médecins Sans Frontières has provided emergency care for tens of thousands of victims of sexual violence in conflict zones, and continues to report that most of these attacks are perpetrated with impunity by militia or military personnel [19]. Amnesty International has documented human rights violations occurring in conflict zones where perpetrators are rarely if ever held accountable, and continually laments the international community's lack of response to sexual violence [22].

While international courts now recognize rape as a war crime, a crime against humanity, a form of torture, and a constituent act of genocide that demands international regulation [9], concerns are mounting that due to lack of preparation and political will, the international criminal court is too frequently dropping charges of sexual violence in their war crimes prosecutions [23]. In addition, there is the deplorable matter of peacekeepers themselves raping women and girls [24,25].

Medical professionals are powerful lobbyists whose recognition of the devastation could galvanize support for the work of humanitarian organizations and advocacy groups in documenting sexual atrocities and holding states accountable when human rights and international law are violated. Together with medical journalists and editors they have done much to try to expose the devastation of sexual violence during conflict [26–28], but we can all do more to document and disseminate the research and accounts of health workers, nongovernmental organizations, and survivors.

A recent set of articles in *PLoS Medicine* [29–31] introduced a new tool called the Dirty War Index (DWI) that can distinguish highly undesirable outcomes of conflict on civilian populations, including rape. The tool's novelty is that it expresses prohibited or undesirable (“dirty”) war outcomes as a *ratio* (rather than an absolute figure). A DWI for war-associated rape could be calculated as: (number raped by combatant group/total number having face-to-face contact with combatant group) × 100. The tool's developers, Madelyn Hsiao-Rei Hicks and Michael Spagat, argue that as ratios, DWIs “lend themselves to comparisons over time, between wars, between weapons, and between warring combatant groups to identify better versus worse performers.” They acknowledge that “war-associated rape may be difficult to measure due to stigma and under-reporting, though substantial reports exist” [29]. A related expert commentary says the DWI “provides a good example of an answer to a recent call for better use of quantitative public health data by conflict analysts and human rights monitors” [31].

Those involved in providing, researching, and reporting medical care can continue to press for support and services for survivors of sexual violence in conflict areas, including emergency contraception, post-exposure prophylaxis, HIV testing, antiretroviral therapy, and counseling. We can lobby to ensure that these efforts to respond medically to sexual violence be linked to broader justice efforts. As part of the care that is imparted to women, for example, we should insist that medical responsibility include supporting survivors to bring charges if they wish, and advocating for international courts to investigate and prosecute perpetrators of sexual violence. But because these prosecutorial efforts are reactive and involve only a small fraction of the perpetrators, they are limited in holding those responsible accountable and in protecting potential victims. Thus our support must also emphasize that justice work be twinned with preventative efforts such that protecting women and girls from sexual violence becomes a central part of peacekeeping and security efforts [21].

Rape as a weapon of war is unconscionable. Medical journalists and editors, along with health care professionals, have the authority, the skills, and the audience to draw the world's attention to the brutality and intolerability of sexual violence in armed conflicts. We need to step up our support of efforts by humanitarian and advocacy organizations to press international authorities to take more concerted actions. Denis Mukwege from Panzi Hospital said in November in a speech in Toronto that it is in every one of us to act to end rape in war. Speaking up is the very least we can do.

## Abbreviations

DRC: Democratic Republic of the Congo
DWI: Dirty War Index
